# Editorial: Online sensory experiences: Consumer reactions to triggering multiple senses by using psychological techniques and sensory-enabling technologies

**DOI:** 10.3389/fpsyg.2022.992114

**Published:** 2022-08-16

**Authors:** Lieve Doucé, Carmen Adams, Olivia Petit, Anton Nijholt

**Affiliations:** ^1^Faculty of Business Economics, Department of Marketing and Strategy, Hasselt University, Diepenbeek, Belgium; ^2^University College PXL, Innovative Entrepreneurship, Hasselt, Belgium; ^3^Kedge Business School, Marketing and New Consumption Center of Excellence, Marseille, France; ^4^Human Media Interaction, University of Twente, Enschede, Netherlands

**Keywords:** online sensory experiences, multisensory environments, sensory-enabling technologies, virtual reality, crossmodal correspondences, extended reality

Over recent years, online shopping has grown at a tremendous rate, reaching a worldwide online retail sales of 4.9 trillion USD in 2021. Moreover, forecasts predict a growth by 50% over the following four years, reaching ~7.4 trillion USD in 2025 (Statista, 2022). Needless to say that it is crucial for retailers to optimally market their offerings online. One disadvantage of the online store environment today is that it mainly uses only two senses (i.e., vision and audition). This shortage of sensory input creates difficulties to present and inspect the products and services comprehensively. For example, previous research stated that a visual representation of a product on a neutral background (e.g., 2D or 3D picture) does not always indicate how this product would look or feel in real life (Javornik, 2016; Hilken et al., 2017; Petit et al., 2022). Furthermore, the online store environment also lacks multisensory atmospheric cues that enables consumers to be fully immersed in a store atmosphere (Petit et al., 2019).

Therefore, there is a need to investigate how the available senses can be used to trigger perceptions *via* the absent senses (e.g., *via* cross-modal correspondences), and this with or without sensory-enabling technologies that can deliver sensory input and enriched experiences (e.g., VR; Petit et al., 2019). This Research Topic aims at widening the knowledge on creating online multisensory customer experiences and comprises four empirical papers, one review on the on- and offline multisensory tasting experience, and one perspective on consumer consciousness in multisensory extended reality.

The first empirical paper assesses the role of different configurations of visual cues commonly present in a product's packaging (jar vs. bag, transparent vs. opaque, labeled vs. unlabeled) in the expectations associated with dietary cookies when presented in e-commerce platforms (Reinoso-Carvalho et al.). From the between-participants study with different combinations of these three features, it can be concluded that changes in the visual appearance of packaging can modulate the expectations consumers have concerning product quality, healthiness, price, and product properties such as sweetness, tastiness, and crumbliness. The presence of labeling and transparent (vs. opaque) packaging yielded higher expectations concerning the quality of the product. The latter, on the other hand, had an opposite effect on the expected healthiness of such cookies. The authors conclude that the visual appearance of packaging can nudge consumers toward healthier purchase habits.

In the second empirical paper the focus is drawn to the inclusion of the olfactory sense since odor perception can be cognitively modulated. In Iseki et al. it is studied whether gender-specific information associated with an otherwise neutral scent may influence congruent olfactory and tactile perception. For example, feminine labeling of a scent could induce a more positive rating of the tactile quality of smooth papers soaked with a neutral scent than when the smell is labeled masculine. From their congruency experiments, they could decide that a feminine-labeled scent was perceived as more feminine than a masculine-labeled scent. However, the feminine and masculine labeling did not influence the haptic perceptions and hedonic evaluations of paper imbued with a neutral scent.

Brengman et al. analyze the effects of scent in multisensory VR ads on consumer experience, depending on whether the scent is coherent or not with the product category. In a 2 ×3 between-subjects study, the researchers manipulate the presence of sound (on vs. off) and scent (congruent vs. incongruent vs. no). They found that whether sound is enabled or not, adding a congruent scent results in a more compelling sensory experience, than without enriching the VR ad with any scent. It should also be noted that adding an incongruent scent (as opposed to no scent) does not result in lower sensory experiences. The authors conclude on the interest of creating multisensory advertising in VR including scents to improve the consumer experience. They also stress the importance of not considering ambient stimuli in isolation, since it is the total configuration of cues that influence consumer responses.

This multisensory perspective is further analyzed by the fourth empirical paper of Doucé et al., who examine multisensory congruency *via* crossmodal correspondences and the moderating role of shopping goals (i.e., experiential vs. goal-directed) on this crossmodal congruency effect. Results show that when the online shop and music played on this webshop are crossmodally congruent (i.e., eliciting the same crossmodal correspondences such as both eliciting the correspondences of cold instead of hot) more positive consumer reactions are present when compared to the crossmodal incongruent situation, irrespective of the shopping goal. When compared to a no music situation, however, the crossmodal congruent situation only has a positive effect for experiential browsers and no adverse effect on goal-directed searchers.

The literature review paper by Spence et al. offers insights into the combined inclusion of the sense of taste and audition. In particular, it investigates the technique of pairing sounds (music) with tastes (flavor), referred to as sonic seasoning. The paper offers insights into the creation of this multisensory tasting experience by reviewing a number of brands and case studies and exploring the added value of this type of sensorial stimulation in the phygital and online environment. In conclusion, the authors make a case for the creation of sonic seasoning whilst taking into account the scientific underlying principles, such as the crossmodal correspondences that may exists between basic tastes and the auditory sense.

In their perspective paper (Petit et al.) discuss the importance of consciousness in the digital consumer experience and how the introduction of multisensory technologies in this environment can challenge this consciousness. How can living experiences through augmented reality, virtual reality, or even in the metaverse represented by an avatar modify the perception of oneself and one's body? How does this affect consumers' consciousness of their experiences, their desires, and choices? Are all questions that are discussed in this paper. The authors conclude on the need for further research to understand how best to stimulate the consumer's senses in digital environments to improve the wellbeing of consumers. They also alert on the ethical issues that the loss of consciousness can generate in these environments.

To conclude, the articles in this Research Topic show that (1) visual and auditory information can influence product expectations (i.e., taste and quality expectations; Reinoso-Carvalho et al.) as well as product perceptions (i.e., scent perceptions: Iseki et al.; taste perceptions: Spence et al.) in other senses, (2) offering a rich online customer experience leading to positive consumer reactions involves a multisensory perspective (i.e., scents added to VR: Brengman et al.; multisensory congruency via crossmodal correspondences: Doucé et al.), and (3) multisensory technologies in a digital customer experience can affect consumers' consciousness (Petit et al.).

## Author contributions

All authors listed have made a substantial, direct, and intellectual contribution to the work and approved it for publication.

## Conflict of interest

The authors declare that the research was conducted in the absence of any commercial or financial relationships that could be construed as a potential conflict of interest.

## Publisher's note

All claims expressed in this article are solely those of the authors and do not necessarily represent those of their affiliated organizations, or those of the publisher, the editors and the reviewers. Any product that may be evaluated in this article, or claim that may be made by its manufacturer, is not guaranteed or endorsed by the publisher.
